# miRNA let-7b modulates macrophage polarization and enhances tumor-associated macrophages to promote angiogenesis and mobility in prostate cancer

**DOI:** 10.1038/srep25602

**Published:** 2016-05-09

**Authors:** Zhigang Wang, Lu Xu, Yinying Hu, Yanqin Huang, Yujuan Zhang, Xiufen Zheng, Shanshan Wang, Yifan Wang, Yanrong Yu, Meng Zhang, Keng Yuan, Weiping Min

**Affiliations:** 1 Institute of Immunotherapy of Nanchang University, and Jiangxi Academy of Medical Sciences, Nanchang, China; 2 Jiangxi Provincial Key Laboratory of Immunotherapy, Nanchang, China; 3 Departments of Surgery, Pathology, and Oncology, University of Western Ontario, London, Canada

## Abstract

Macrophage polarization is a highly plastic physiological process that responds to a variety of environmental factors by changing macrophage phenotype and function. Tumor-associated macrophages (TAMs) are generally recognized as promoting tumor progression. As universal regulators, microRNAs (miRNAs) are functionally involved in numerous critical cellular processes including macrophage polarization. Let-7b, a miRNA, has differential expression patterns in inflamed tissues compared with healthy controls. However, whether and how miRNA let-7b regulates macrophage phenotype and function is unclear. In this report, we find that up-regulation of let-7b is characteristic of prostatic TAMs, and down-regulation of let-7b in TAMs leads to changes in expression profiles of inflammatory cytokines, such as IL-12, IL-23, IL-10 and TNF-α. As a result, TAMs treated with let-7b inhibitors reduce angiogenesis and prostate carcinoma (PCa) cell mobility. Let-7b may play a vital role in regulating macrophage polarization, thus modulating the prognosis of prostate cancer.

Prostate carcinoma (PCa) with its associated morbidity and mortality continues to be an important health issue in males worldwide. In 2016, 180,890 new prostate cancer cases and 26,120 deaths are projected to occur in the United States^1^. Morbidity and mortality rates are rising in other countries as a result of the increased aging population. Cancer tissue and the surrounding stromal cells compose the tumor microenvironment that provides opportunity for reciprocal interactions among cancer cells, inflammatory cells and microcapillary vessels. Inflammation is linked clinically and epidemiologically to cancer, and tumor-associated macrophages (TAMs) appear to play a causative role, but the mechanisms are poorly understood.

Macrophages have an indispensable role in the immune system with decisive functions in both innate and acquired immunity. Despite resident macrophages providing immediate defence against foreign pathogens and coordinating leukocyte infiltration in innate immunity, the presence of TAMs within the tumour microenvironment has been associated with enhanced tumor progression including cancer cell growth and spread, angiogenesis and immune suppression^2^,^3^,^4^,^5^. This paradoxical role of macrophages in cancer can be explained by their functional plasticity. Two nomenclatures have been used to describe macrophage phenotypes. Classical or type 1 (M1) macrophage activation, in response to microbial products or interferon-γ (IFN-γ), is characterized by its capacity to present antigen and increase IL-12 and IL-23 production with subsequent activation of a polarized type I response. Alternatively, activated or type 2 (M2) macrophages are induced by type 2 cytokines, such as IL-4 and IL-13, and characterized by an IL-10^high^ and IL-12^low^ phenotype. M1 macrophages are generally considered potent effector cells that kill microorganisms and tumor cells and produce copious amounts of pro-inflammatory cytokines, whereas M2 macrophages fine-tune inflammatory responses and adaptive Th2 immunity, scavenge debris, and promote angiogenesis, tissue remodeling and repair^6^,^7^. Increased infiltration of TAMs has been associated with worse pathological characteristics and poor prognosis in breast, colon, and bladder cancers^8^,^9^. Similarly, TAM infiltration in prostate biopsy specimens is a useful predictive factor for prostate-specific antigen (PSA) failure or progression of PCa after hormonal therapy^10^. Recently, Gollapudi and colleagues^11^ demonstrated that cancer cores and high-grade prostate carcinoma had more TAMs than prostatic intraepithelial neoplasia and benign tissue, suggesting the potential role of TAMs in PCa development.

MicroRNAs (miRNAs) are already known as master epigenetic regulators. It is estimated that 30–90% of human genes are regulated by miRNAs, which makes these molecules of great importance for cell growth, activation, apoptosis and differentiation. The let-7 family, one of miRNAs, regulates developmental timing and cell proliferation, mediates immune responses, and adjusts inflammation^12^,^13^. Previous studies have supported that let-7 family is associated with human cancers as it is significantly decreased in a variety of tumors^14^,^15^. In addition, decreased expression of let-7b in PCa cells has been linked to a higher risk of PCa prognosis^16^. Recent studies have shown that let-7f, another member of the let-7 family, was over-expressed in tuberculosis-infected macrophages that induce tumor necrosis factor (TNF), and IL-1β secretion. This process is regulated by A20, which is the target of let-7 and an inhibitor of the NF-κB pathway^17^. Let-7 regulates expression of some cytokines such as IL-6 and IL-10, yet its significance in TAMs derived from PCa remains unclear.

In the present study, we investigated the role of let-7-mediated macrophage polarization in PCa progression. We identified that let-7 modulates cytokine profiles in PCa-conditioned TAMs, allowing the setup of a pro-inflammatory or pro-tumor microenvironment. In turn, these inflammatory cytokines actively affected motility and angiogenesis of PCa cells, ultimately fostering cancer cells’ escape from primary tumors and favoring metastatic spread.

## Results

### Generation and characterization of PCa-conditioned TAMs

**Figure 1:**
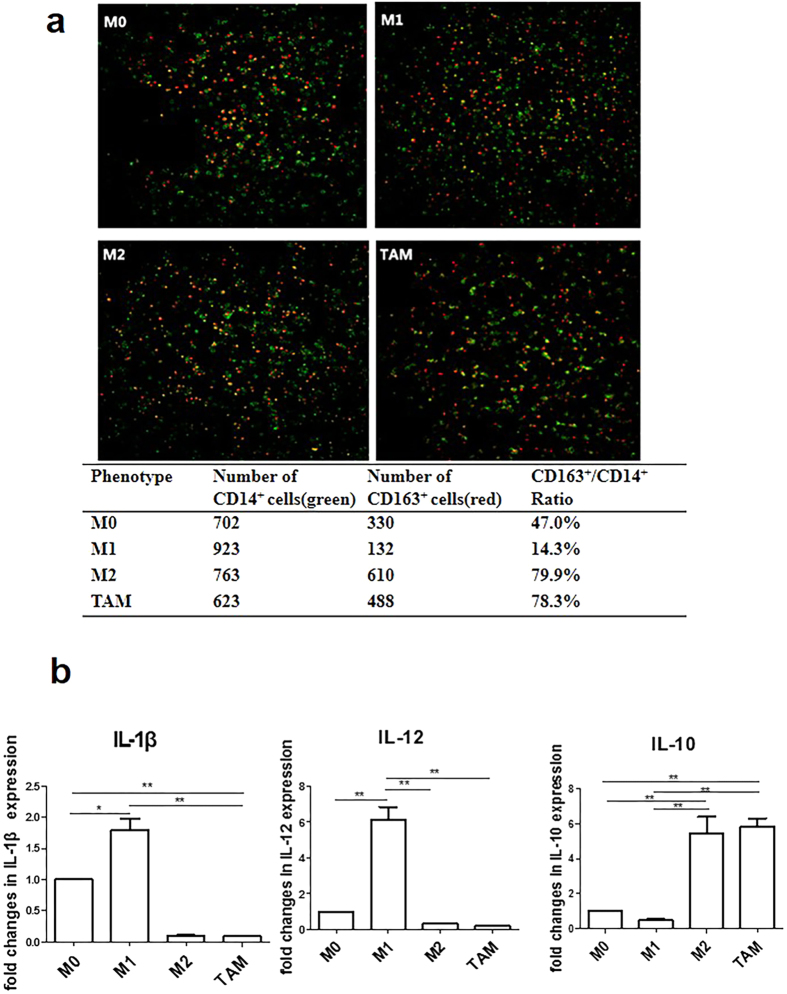
TAMs, induced by CM from PC-3, show characteristics of M2-like phenotype. (**a**) CD163/CD14 ratio in TAMs. Human monocytes were isolated from normal donor buffy coat using anti-CD14 magnetic beads. Monocytes were cultured in the presence of M-CSF for 7 days. TAMs, M1 and M2 macrophages were differentiated as described in the Materials and Methods. Characterization of subtypes of macrophages with anti CD-14 FITC (green) and anti CD-163 PE (red) was detected by High Throughput Connotation of Imaging System. (**b**) Cytokines profiles. After induction of TAMs, M0, M1 and M2 macrophages, levels of IL-12, IL-1β and IL-10 were measured by real-time RT-PCR. The expression of mRNAs was normalized to GAPDH. *P < 0.05; **P < 0.01.

Macrophages are highly plastic cells that respond to a variety of environmental cues by changing their phenotype and function^18^. Proinflammatory M1 macrophages promote T helper (Th) 1 responses and show tumoricidal activity. M2 macrophages display regulatory functions in tissue repair and remodelling and promote Th2 immune responses^19^. TAMs, key orchestrators in the tumor microenvironment, resemble M2-polarized macrophages. Circulating monocytes, which can pass through the vascular endothelium to mature into macrophages in the peripheral tissues, are activated in various ways by endogenous and exogenous factors^20^. To investigate if exposure to PCa tumor microenvironment can affect monocyte differentiation, we incubated human blood monocytes (isolated from healthy male donors) with culture medium (CM) collected from PCa cells for 7 days. Afterwards, we detected the phenotype of the macrophages. First, we analyzed expression of CD163, a marker for M2 in TAMs. TAMs expressed almost the same level of CD163 (78.3%) as M2 (79.9%) (Fig. 1A). Next, we measured the expression of IL-10, IL-12 and IL-1β which have been used for phenotyping macrophages. Our data show that TAMs displayed IL-10^high^, IL-12^low^, and IL-1β^low^ phenotype (Fig. 1B). These data indicate that PCa-conditioned prostatic TAMs possess M2-like phenotype.

### PCa-conditioned TAMs enhance tumorigenesis of PC-3 cells and promote angiogenesis of endothelial cells

**Figure 2:**
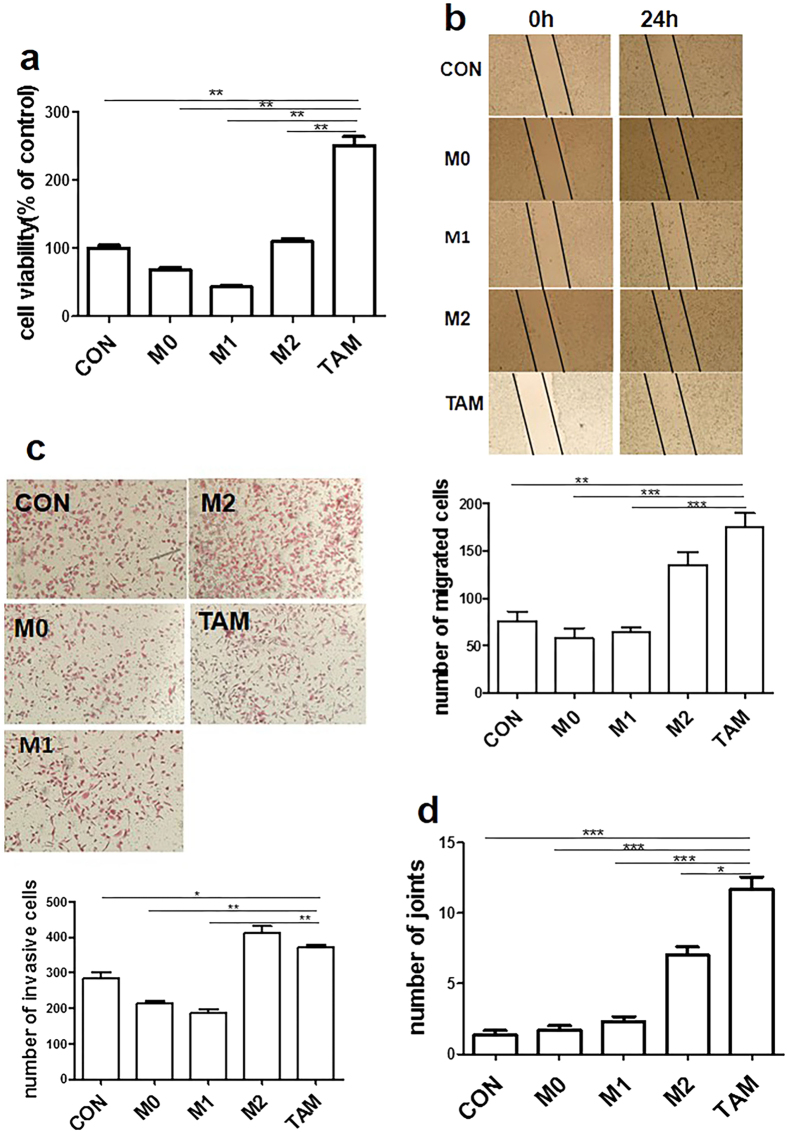
TAMs promote proliferation, mobility and invasiveness of PC-3 cells and promote angiogenesis. (**a**) Proliferation of PC-3 cells. PC-3 cells were exposed to CM from M0, M1, M2 and TAMs or 1640 medium as control for 72 h. Viability of PC-3 cells was measured by MTT assay. (**b**) Mobility of PC-3 cells. A line of PC-3 cells was scraped away in each well using a pipette tip after 6 h of serum starvation. Subsequently, cells were treated with CM from M0, M1, M2 and TAMs for 24 h. Migrated cells were observed from three randomly chosen fields (original magnification, 100×) and the number of migrated cells was quantified by manual counting. (**c**) Invasiveness of PC-3 cells. PC-3 cells were loaded into the upper compartments and then placed into 24-well culture dishes containing different CM from M0, M1, M2 and TAMs, or RPMI 1640 medium as control. After 24 h of incubation at 37 °C, cells that migrated to the bottom of the membrane were stained with hematoxylin-eosin and counted using an inverted microscope (original magnification, 100×). (**d**) Tube-like structure formation in HUVEC. HUVEC were seeded to the matrigel-coated plates, followed by addition of CM from M0, M1, M2 or TAMs, respectively. The effects on the morphogenesis of endothelial cells were recorded after 5 h with an inverted microscope equipped with CCD optics and a digital analysis system. Results were quantified by measuring the joint or vessel numbers in the field (original magnification, 100×). *P < 0.05; **P < 0.01; ***P < 0.001.

In solid tumors, TAMs correlate with high vessel density and tumor progression. Accumulated evidence demonstrates that TAMs play a critical role in the regulation of epithelial-mesenchymal transition in cancer. To validate the role of PCa-conditioned TAMs, we studied their effect on PC-3 cell proliferation, migration and invasion. The results show that PCa-conditioned TAMs significantly enhance PC-3 cell proliferation (Fig. 2A), migration (Fig. 2B) and invasion (Fig. 2C). We also determined their ability to promote angiogenesis. As shown in Fig. 2D, prostatic TAMs were pro-angiogenesis, a powerful provocation compared to other macrophage subtypes, although they showed characteristics of M2-like phenotype.

### Up-regulation of miRNA let-7b in PCa-conditioned prostatic TAMs

**Figure 3:**
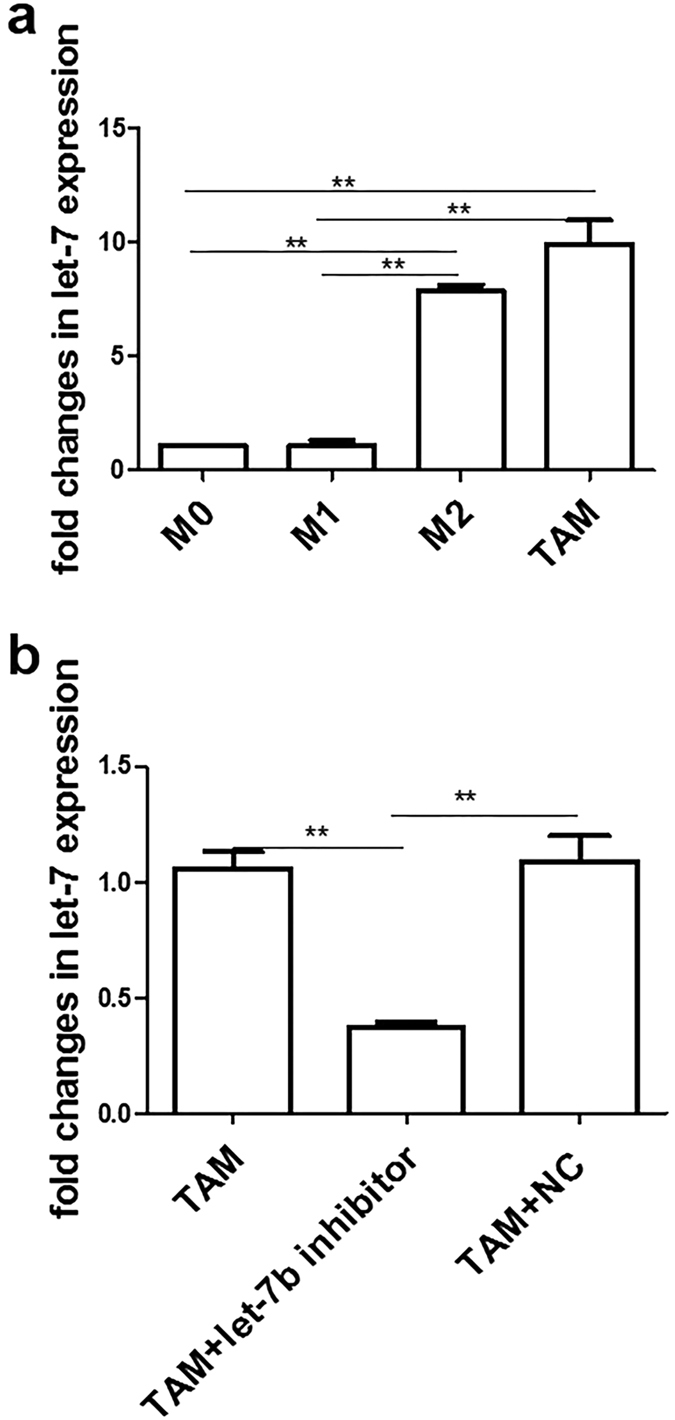
Up-regulation of let-7b expression in prostatic TAMs. (**a**) Let-7b expression in different macrophages. M0, M1, M0 and TAMs were differentiated as described in Materials and Methods. Let-7b expression was determined by real-time RT-PCR. Data represent mean ± SD of three independent RT-PCR results. The expression of miRNA was normalized to U6. (**b**) Expression of let-7b is down-regulated in TAMs treated by its inhibitor. TAMs were transfected with let-7b inhibitors, negative control (NC), or remained untreated for 72 h. Relative expression of let-7b was analyzed by real-time RT-PCR. The expression of miRNA was normalized to U6. Data represent mean ± SD of three independent experiments. *P < 0.05; **P < 0.01.

To investigate the mechanism by which TAMs could affect cytokine expression, we first detected the expression of let-7b. We found that prostatic TAMs significantly increased expression levels of let-7b compared with other macrophages (Fig. 3A). Next, we transfected TAMs with either let-7b inhibitors or negative control of inhibitors. We observed that let-7b was significantly down-regulated by as much as 37.12% with let-7b inhibitors but not with negative control of inhibitors (Fig. 3B), suggesting upregulated let-7b in PCa-conditioned TAMs can be efficiently suppressed by let-7b inhibitors.

### Let-7b regulates expression of inflammatory cytokines in PCa-conditioned TAMs

**Figure 4:**
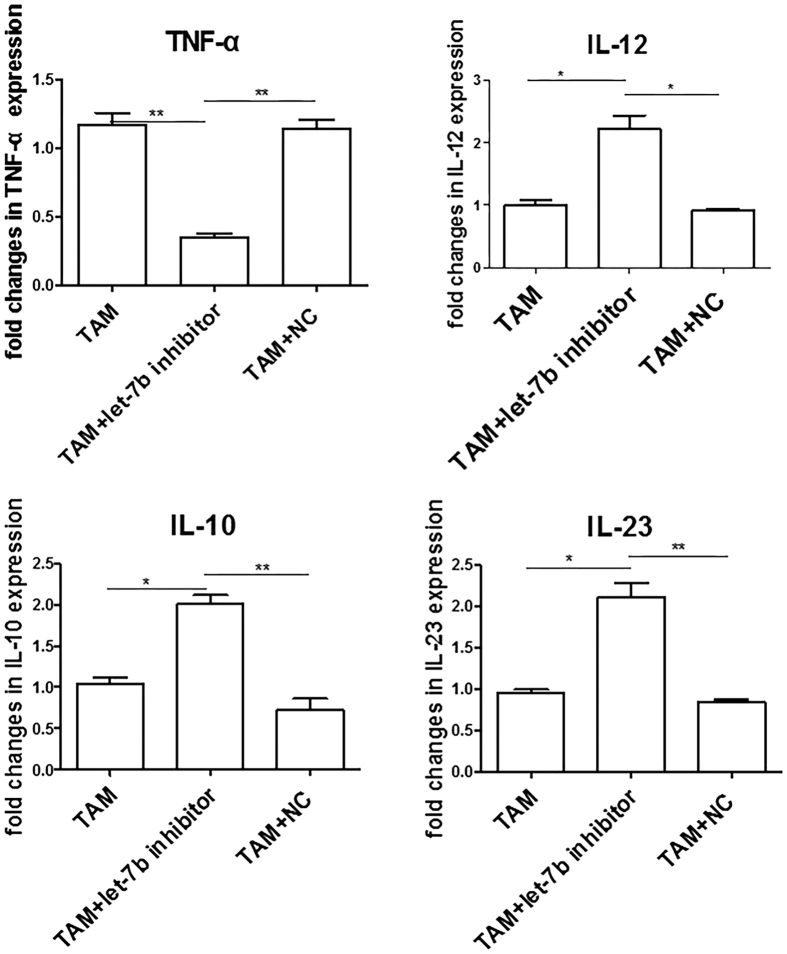
Let-7b regulates expression of inflammatory cytokines in TAMs. TAMs were differentiated as described in Materials and Methods. TAMs were transfected with let-7b inhibitors, negative control (NC), or remained untreated for 72 h. Expression of IL-12, IL-23, IL-10 and TNF-α was determined using real-time RT-PCR. Expression of mRNAs was normalized to GAPDH. Data represent mean ± SD of three separate experiments. *P < 0.05; **P < 0.01.

TAMs, acting as tumorigenesis regulators, work partially through secretion of pro-inflammatory cytokines (such as TNF-α and IL-12) or anti-inflammatory cytokines (such as IL-10)^21^. To further investigate the role of let-7b in TAMs, we analyzed expression of inflammatory cytokines including IL-12, IL-23, IL-10 and TNF-α. Our results reveal that, in the presence of let-7 inhibitors, IL-12 and IL-23 were significantly up-regulated whereas TNF-α was down-regulated in PCa-conditioned TAMs. Interestingly, IL-10, a cytokine related to M2, was also significantly increased by let-7b inhibitors (Fig. 4). These data indicate that let-7b modulates the expression of IL-12, IL-23, IL-10 and TNF-α in PCa-conditioned TAMs.

### Let-7b promotes mobility of PC-3 cells

**Figure 5:**
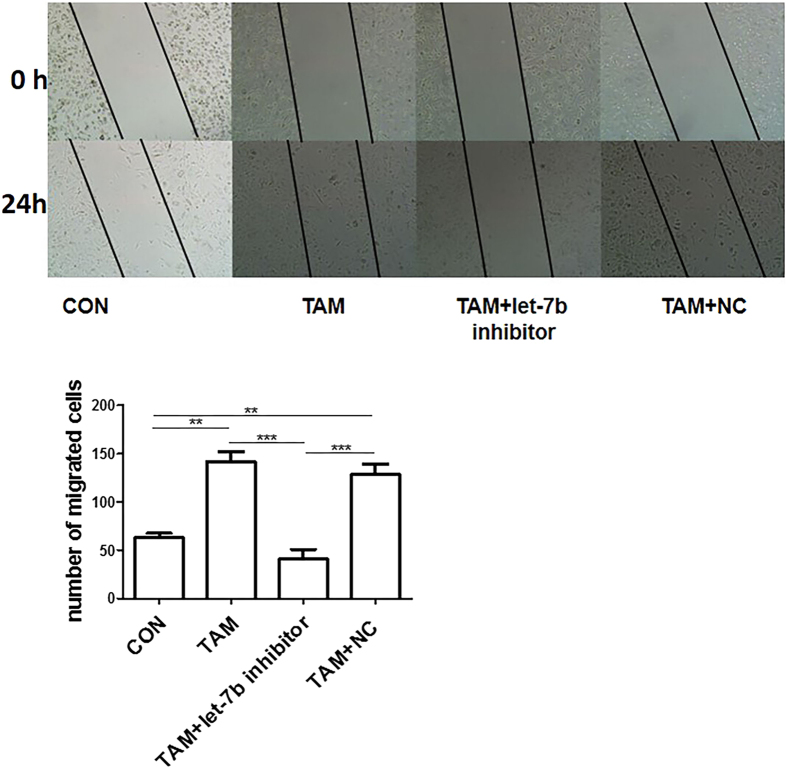
TAMs treated with let-7b inhibitor decrease PC-3 mobility. PC-3 cells were cultured and scraped away using a pipette tip. Subsequently, cells were cultured with CM from TAMs treated with let-7b inhibitors, negative control (NC), or RPMI 1640 medium (control) for 24 h. The migrated cells were observed from three randomly chosen fields (original magnification, 100×) and quantified by manual counting. *P < 0.05; **P < 0.01; ***P < 0.001.

Cell migration is essential for diffusion of cancer cells during PCa progression^22^. We had observed that let-7b influences expression of inflammatory cytokines in TAMs. To clarify if introduction of let-7b inhibitors into TAMs could impair PCa migration, we incubated human PCa cells with CM from TAMs that had been transfected with let-7b inhibitors or negative control. In a cell migration assay, we observed that let-7b inhibitors significantly suppressed the capacity of PCa-conditioned TAMs to promote PC-3 migration as compared with negative control or TAMs without transfection (Fig. 5). These results suggest that let-7b expression in TAMs plays a critical role in promoting PCa migration.

### Let-7b enhances pro-angiogenesis of PCa-conditioned TAMs

**Figure 6:**
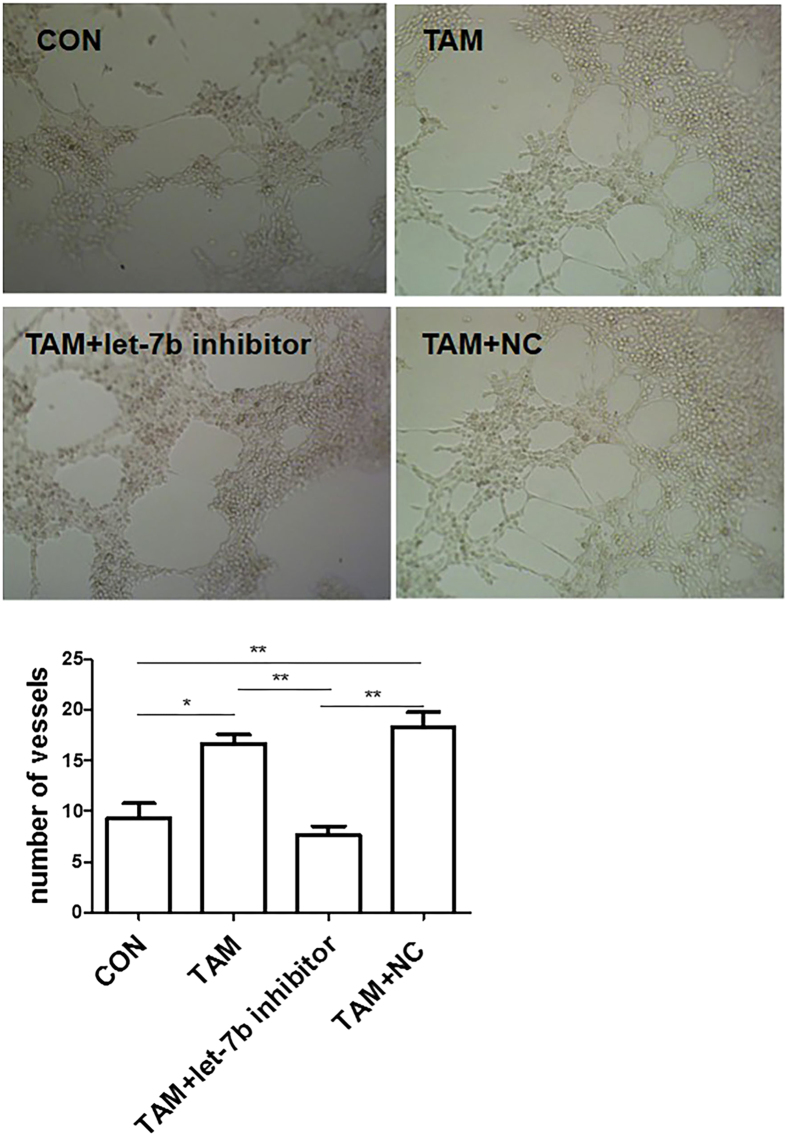
Down-regulation of let-7b suppresses pro-angiogenic effect of TAMs. HUVEC were plated in a 96-well plate pre-coated with matrigel, followed by the addition of CM from TAMs treated with let-7b inhibitors, negative control (NC), or RPMI 1640 medium (control) for 5 h. The effects on the morphogenesis of endothelial cells were imaged with an inverted microscope equipped with CCD optics and a digital analysis system. Results were quantified by measuring the joint or vessel numbers in the field (original magnification, 100×). *P < 0.05; **P < 0.01.

An important step during neo-angiogenesis is the formation and merging of tubes, produced by endothelial cells, to form a complex network of vessels and capillaries^23^. To determine the effect of let-7b on pro-angiogenesis of PCa-conditioned TAMs, we used human umbilical vein endothelial cells (HUVEC) as they have been reported to drive *de novo* angiogenesis in tube-like structure formation^24^. We treated HUVEC with CM from PCa-conditioned TAMs transfected with let-7b inhibitors, then assessed their ability to induce capillary morphogenesis. As shown in Fig. 6, let-7b inhibitors significantly suppressed tube-like structure formation, suggesting let-7b has a key role in driving vascularization of PCa.

## Discussion

Let-7 family members, specifically let-7b, have been implicated as tumor suppressors in several types of human tumor cells including prostate carcinoma^25^. However, little has been reported on the role of let-7b in prostatic TAMs. In this study, we demonstrate the importance of let-7b, confirming that its decreased expression inhibits the pro-angiogenic effect of TAMs and their capacity to enhance PC-3 cell motility. The expression level of let-7b significantly and positively correlates to the level of TNF-α, while let-7 negatively regulates the expression of IL-10, IL-12 and IL-23 in TAMs.

miRNAs are universal regulators of differentiation, activation and polarization of macrophages. A number of studies have implicated different miRNAs in human monocytes/macrophages in response to inflammatory stimuli^26^,^27^. Our former studies had demonstrated that the level of let-7a/b/c/e was upregulated in TAMs as comparing with other macrophages, and among let-7 miRNAs, let-7b showed the most obvious difference (unpublished data). However, the role of let-7b in regulating macrophage polarization has been largely undefined. We found that let-7b is expressed in prostatic TAMs at the highest level, comparable to M0, M1 and M2 macrophages. Our data show that let-7b is involved in macrophage polarization and affects function of TAMs. Down-regulation of let-7b in TAMs significantly suppresses PCa migration and the function of pro-angiogenesis. One plausible explanation is that let-7b regulates a variety of inflammatory cytokines, leading to the change in biological properties of TAMs. Known relevant targets of let-7b are molecules involving cell cycle control with respect to differentiation and tumorigenesis like Estrogen Receptor-α36, HMGA1, EZH2 and Cdc34^16^,^28^,^29^,^30^. Recent studies found that let-7 regulates C/EBP-δ, an important transcriptional factor that has been shown to be required for a sustained TLR4 signals which induced NF-κB and AP-1 activation^31^,^32^. Targeting of the SOCS4 3’ untranslated region by let-7b resulted in translational repression and inhibition of SOCS4 promoted phosphorylation of STAT3 and STAT6^33^. These signal molecules regulate the secretion of a large number of cytokines such as IL-6 and IL-12. Experimental data indicate that the let-7 family members suppress several important immune-related genes including IL-6, IL-13 and IL-10^34^,^35^,^36^. In support of this theory, our data show that transfection with let-7 inhibitors can change pro- and anti-inflammatory cytokine profiles.

All solid tumors require the induction of new blood vessels to grow, and angiogenesis is associated with tumor growth and metastasis. Microvessel density in the area of the most intense neovascularization in invasive, early-stage breast carcinoma is an independent and highly significant prognostic indicator for overall and relapse-free survival in patients^37^. At least two general categories are recognized: (i) angiogenic activity arises from the tumor cell itself by releasing angiogenic molecules such as basic fibroblast growth factor; (ii) angiogenic activity arises from host cells recruited by the tumor (e.g. macrophages)^23^. Cell motility is a fundamental component of many physiological and pathological processes and drives disease progression in cancer^22^. It is a critical step in the progression of PCa to the metastatic state, the lethal form of the disease.

TAMs have been associated with enhanced tumor progression, including cancer cell growth and spread, angiogenesis and immune suppression. In this study, human monocytes became prostatic TAMs after incubation with CM collected from PC-3 cell culture. The resultant TAMs displayed characteristics of M2-like macrophages, such as CD163^high^, IL-10^high^ and IL-12^low^. We found that TAMs significantly enhance PC-3 cell proliferation when compared to control, M0, and M1. Moreover, we found that transfection of TAMs with let-7 inhibitors decreases the level of let-7b and reverses the effect of TAMs on PCa migration and angiogenesis.

Cytokines in the tumor microenvironment have the capacity to pilot recruitment, maturation and differentiation of infiltrating leukocytes, playing a vital role in the growth and metastasis of tumor cells. Our data demonstrate that IL-10, IL-12 and IL-23 are significantly up-regulated in TAMs treated with let-7b inhibitors, whereas TNF-α shows significant down-regulation. IL-12 and IL-23 are predominantly pro-inflammatory/pro-stimulatory cytokines that have key roles in the development of Th1 and Th17 cells, respectively^38^. Considering the M1 phenotype produces IL-12 and IL-23, we hypothesized that TAMs treated with let-7b inhibitors would display partial characteristics of M1 with IL-12^high^ and IL-23^high^ phenotype. Accumulated evidence indicates that IL-12 is a cytokine with both immunostimulatory and antiangiogenic effects^39^,^40^,^41^,^42^. The mechanism of IL-12’s antitumor action may depend, not only on the immunostimulatory activity of this cytokine, but also on its effect on tumor cell angiogenesis^43^. IL-10 was also significantly up-regulated in TAMs treated with let-7b inhibitors. One explanation is that IL-10 is a target of let-7b, and its expression is negatively mediated by let-7b^35^. With decreased expression of let-7b, mRNA stability of IL-10 increases in TAMs treated by an inhibitor. Although IL-10 has been reported as a cytokine related to M2, IL-10 is drawing attention as an inhibitor of tumor angiogenesis^44^. Kohno and colleagues have reported anti-angiogenic and tumor suppressive effects of IL-10 in ovarian cancer cells^45^. In addition, IL-10 also inhibits cell mobility^46^,^47^. In our data, down-regulation of let-7b leads to significant up-regulation of IL-10, IL-12 and IL-23 in TAMs. We speculate that these cytokines synergize to suppress angiogenesis. We also found that TNF-α is significantly down-regulated in TAMs treated with let-7b inhibitors. TNF superfamily cytokines are increasingly recognized as key modulators of angiogenesis. TNF-α is not only involved with angiogenesis^48^,^49^,^50^,^51^, but also enhances the motility and invasiveness of prostatic cancer cells^52^. It is possible that down-regulation of TNF-α, mediated by let-7b, contributes to the suppression of angiogenesis and PC-3 mobility.

In summary, our results indicate that the tumor-promoting role of prostatic TAMs may be partially ascribed to up-regulation of let-7b, which regulates expression of IL-12, IL-23, IL-10 and TNF-α. The expression profiles of these inflammatory cytokines partially affect TAM function. TAMs that were treated with let-7b inhibitors had reduced angiogenesis and PCa cell mobility. It should be noted that let-7b may possess various functions owing to its pleiotropic regulation of genes. It is our expectation that additional let-7b target genes will be identified in the near future. Our findings suggest that let-7b is a promising modulator for macrophage polarization. Further studies are required on the origin of let-7b up-regulation and its signal pathway to determine if let-7b can be used to mediate macrophage polarization.

## Materials and Methods

### Isolation and culture of human peripheral blood macrophages

Blood monocytes were isolated from healthy donor buffy coats. Peripheral blood mononuclear cells (PBMCs) were isolated using a Ficoll (Solarbio Life Sciences, Beijing, China) density gradient, and subsequently monocytes were isolated from PBMCs using anti-CD14 magnetic beads (Miltenyi Biotec, Bergisch Gladbach, Germany) according to the manufacturer’s protocol. Non-adherent cells were removed, and purified monocytes were incubated for 7 days in RPMI 1640 (Life Technologies Corporation, Grand Island, NY, USA), supplemented with 10% FBS (Life Technologies, Burlington, ON, Canada) and 50 ng/ml M-CSF (Peprotech, Rocky Hill, NJ, USA) to obtain macrophages. M0 cells were obtained by treating with serum-free medium for 48 h. M1 macrophages were polarized by stimulating overnight with 100 ng/ml lipopolysaccharides (Peprotech) and 100 ng/ml IFN-γ (Peprotech). M2 macrophages were polarized by stimulating overnight with 20 ng/ml IL-4 (Peprotech). Prostatic TAMs were obtained by culturing monocytes for 7 days in RPMI 1640 10% FBS with 50% of conditioned medium (CM) from PC-3 cells. Before CM was obtained from PC-3 cells, M0, M1, M2 and TAMs, these cells were incubated for 48 h in serum- starved condition. Then CM was harvested, clarified by centrifugation and used freshly. The study was approved by the Ethics Committee of the First Affiliated Hospital of Nanchang University, and written informed consent was obtained from all donors. All experimental protocols were in accordance with the approved guidelines for safety requirements of Jiangxi Academy of Medical Sciences, Nanchang University.

### Macrophage marker expression

CD14+ monocytes (2 × 10^5^ cells in 100 μl) were seeded in a 96-well plate. After activation of the macrophages for 48 h, supernatant was collected. Cells were washed twice with PBS. Subsequently, the macrophages were labeled with primary antibody (CD14-FITC, CD163-PE as M2 marker) (eBioscience, San Diego, CA, USA) for 10 min at room temperature. Then stained cells were washed with PBS twice, and 1640 medium was added. The expression of CD-14 and CD-163 was detected by High Throughput Connotation of Imaging System (Molecular Devices, Silicon Valley, CA, USA).

### *In vitro* tumor cell invasion assay

Cell invasion was assayed with the Transwell system of Costar (Corning Incorporated, Corning, NY, USA) equipped with 8-mm-pore size. Matrigel (BD Biosciences, San Jose, CA, USA) was added to the top chamber, then solidified for 1 min at 37 °C and air dried. The matrigel barrier was rehydrated with 100 μl of 1640 for 2 h at 37 °C prior to use. PC-3 cells (ATCC, Manassas, VA, USA) were loaded into the upper compartment (2 × 10^5^ cells in 200 μl) in serum-deprived growth medium. The chambers then were placed into 24-well culture dishes containing 500 μl of CM from different cells. After 24 h of incubation at 37 °C, non-invading cells and the matrigel layer were carefully removed using cotton swabs, and the microporous membrane containing the invaded cells was fixed in 4% PFA (Sigma-Aldrich, St. Louis, MO, USA) and stained with hematoxylin-eosin staining solutions. The ability of PC-3 invasion was evaluated by counting the cells that migrated to the lower surfaces of the polycarbonate filters.

### MTT (3-(4,5-Dimethylthiazol-2-yl)-2,5-diphenyltetrazolium bromide) assay

PC-3 cell viability was measured by MTT assay. Briefly, PC-3 cells were seeded at 5 × 10^3^ cells/well in 96-well plates, and allowed to adhere to obtain 80% confluent monolayer. The medium was replaced with CM from different cells. After 72 h incubation, cell growth was measured at 490 nm using SpectraMax M4 Multimode Microplate reader (Molecular Devices). The number of viable cells was presented relative to control group.

### RNA extraction and real-time reverse transcription PCR

Total RNA was extracted using Invitrogen Trizol Reagent (Life Technologies Corporation). For miRNA quantification, 100 ng total RNA was reverse transcribed directly using stem-loop primers^53^. For mRNA analyses, cDNAs were synthesized from 2 μg total RNA, using oligo (dT) 15 primers and Moloney Murine Leukemia Virus Reverse Transcriptase (Life Technologies Corporation). Quantitative real-time PCR was performed using the SYBR Green PCR Master Mix (Tokara, Kyoto, Japan) in a final volume of 20 μL on Bio-RAD CFX96^TM^ Real-Time System (Bio-Rad Laboratories, Inc., Hercules, CA, USA). The expression of miRNA and mRNAs was normalized to U6 and GAPDH, respectively. Data are presented as relative quantification based on the calculation of 2^−ΔΔCt^. All primers used in this study are shown in change to Supplementary Dataset.

### Matrigel angiogenesis assay

50 μl of matrigel was added to each well of a 96-well plate, then placed in a incubator at 37 °C for 30 min. HUVEC (ATCC) (2 × 10^4^ cells/well) were added to the matrigel-coated plates in a final volume of 100 μl. The effects on morphogenesis of endothelial cells were recorded with an inverted microscope equipped with CCD optics and a digital analysis system (Olympus, Tokyo, Japan) 5 h later. Results were quantified by measuring the joint or vessel numbers in the field.

### Cell migration assay

PC-3 cells were plated and allowed to grow to full confluence in 24-well plates. A line of PC-3 cells was scraped away in each well using a pipette tip 6 h after serum starvation. Subsequently, cells were washed twice to remove detached cells. Fresh 1640 or CM from different cells was added to the scratched monolayers. Images were taken using Olympus 1 × 71 microscope (Olympus, Tokyo, Japan) 24 h after incubation. The migrated cells were observed from three randomly chosen fields and quantified by manual counting^54^.

### Transfection assay

The chemically-modified hsa-let-7b inhibitors and the corresponding negative control oligonucleotides were purchased from RiboBio Corporation (Guangzhou, China). TAMs were transfected with 100 nM let-7b inhibitors, 100 nM negative control (NC) or remained untreated using FuGENE^®^ 6 Transfection Reagent (Promega Corporation, Madison, Wisconsin, USA) according to the manufacturer’s instructions. The cells were harvested after 72 h of transfection for subsequent experiments.

### Statistics

Data are presented as mean ± SD and analyzed using GraphPad Prism 5 (GraphPad Software Inc., San Diego, CA, USA). In cases of multiple tests, one-way ANOVA followed by Bonferroni-Holm procedure was applied. P values < 0.05 were defined as statistically significant.

## Additional Information

**How to cite this article**: Wang, Z. *et al*. miRNA let-7b modulates macrophage polarization and enhances tumor-associated macrophages to promote angiogenesis and mobility in prostate cancer. *Sci. Rep.* **6**, 25602; doi: 10.1038/srep25602 (2016).

## Supplementary Material

Supplementary Information
