# Supplementary material for: miRNA let-7b modulates macrophage polarization and enhances tumor-associated macrophages to promote angiogenesis and mobility in prostate cancer
Source: Sci Rep. 2016 May 9;6:25602. doi: 10.1038/srep25602 (PMC4860600; doi:10.1038/srep25602)
Supplement: Supplementary Information [file srep25602-s1.doc]

**miRNA let-7b modulates macrophage polarization and enhances tumor-associated macrophages to promote angiogenesis and mobility in prostate cancer**

Zhigang Wang^1,2^, Lu Xu^1,2^, Yinying Hu^1,2^, Yanqin Huang^1,2^, Yujuan Zhang^1,2^ Xiufen Zheng^3^, ShanshanWang^1,2^, Yifan Wang^1,2^, Yanrong Yu ^1,2^, Meng Zhang^1,2^, Keng Yuan^1,2^, Weiping Min^1,2,3*^.

1. Institute of Immunotherapy of Nanchang University, and Jiangxi Academy of Medical Sciences, Nanchang, China; 2. Jiangxi Provincial Key Laboratory of Immunotherapy, Nanchang, China; 3. Departments of Surgery, Pathology, and Oncology, University of Western Ontario, London, Canada

**Running Title:** Let-7b modulates TAMs in prostate cancer

***Correspondence** should be addressed to Dr Weiping Min, Email: weiping.min@gmail.com

Table1. A list of all the primers

| Primer name |  | Primer sequences |
| --- | --- | --- |
| GAPDH forward primer  GAPDH reverse primer  TNF-alpha forward primer  TNF-alpha reverse primer  IL-12 forward primer  IL-12 reverse primer  IL-10 forward primer  IL-10 reverse primer  IL-23 forward primer  IL-23 reverse primer  U6 forward primer  U6 reverse primer  let-7b stem-loop Primer  let-7b forward primer  let-7b reverse primer | | 5′-GACCCCTTCATTGACCTCAAC-3′  5′-CTTCTCCATGGTGGTGAAGA-3′  5′-AAAACAACCCTCAGACGCCACATC-3  5′-ACGTCCCGGATCATGCTTTCAGT-3  5'-AGTGGAGTGCCAGGAGGACA-3'  5'-TTCTTGGGTGGGTCAGGTTT-3'  5'-CATCAGGGTGGCGACTCTAT-3'  5'-TGGGCTTCTTTCTAAATCGTTC-3'  5'-CTCAGGGACAACAGTCAGTTC-3'  5'-ACAGGGCTATCAGGGAGCA-3  5′-AAAATATGGAACGCTTCACGAA-3′  5′-GTGCTCGCTTCGGCAGCACATAT-3′  5′-GTCGTATCCAGTGCAGGGTCCGAGGTG  CACTGGATACGACCACCCACCAACCAC-3′  5'-TGAGGTAGTAGGTTGTGTGGTT-3'  5'-GTGCAGGGTCCGAGGT-3 |
